# Deep HDR Deghosting by Motion-Attention Fusion Network

**DOI:** 10.3390/s22207853

**Published:** 2022-10-16

**Authors:** Yifan Xiao, Peter Veelaert, Wilfried Philips

**Affiliations:** Department of Telecommunications and Information Processing, IPI-IMEC, Ghent University, 9000 Ghent, Belgium

**Keywords:** high dynamic range imaging, image fusion, convolutional neural network, attention module

## Abstract

Multi-exposure image fusion (MEF) methods for high dynamic range (HDR) imaging suffer from ghosting artifacts when dealing with moving objects in dynamic scenes. The state-of-the-art methods use optical flow to align low dynamic range (LDR) images before merging, introducing distortion into the aligned LDR images from inaccurate motion estimation due to large motion and occlusion. In place of pre-alignment, attention-based methods calculate the correlation between the reference LDR image and non-reference LDR images, thus excluding misaligned regions in LDR images. Nevertheless, they also exclude the saturated details at the same time. Taking advantage of both the alignment and attention-based methods, we propose an efficient Deep HDR Deghosting Fusion Network (DDFNet) guided by optical flow and image correlation attentions. Specifically, the DDFNet estimates the optical flow of the LDR images by a motion estimation module and encodes that optical flow as a flow feature. Additionally, it extracts correlation features between the reference LDR and other non-reference LDR images. The optical flow and correlation features are employed to adaptably combine information from LDR inputs in an attention-based fusion module. Following the merging of features, a decoder composed of Dense Networks reconstructs the HDR image without ghosting. Experimental results indicate that the proposed DDFNet achieves state-of-the-art image fusion performance on different public datasets.

## 1. Introduction

The limited capabilities of ordinary digital camera sensors make it challenging to reproduce scenes accurately with high dynamic ranges (HDR). Professional HDR cameras can obtain HDR images. However, they are prohibitively expensive and difficult to use for the average consumer [1]. Exposure bracketing techniques, such as multi-exposure image fusion, address this computationally by capturing multiple images of the same scene at different exposure levels and then fusing them. Multi-exposure image fusion can provide high-quality HDR images at a low cost and is therefore widely used in the consumer electronics field [2].

In traditional multi-exposure fusion methods for static scenes [3], all low dynamic range (LDR) images are assumed to be perfectly aligned. However, moving objects are unavoidable when taking LDR images, resulting in ghosting effects after applying multi-exposure image fusion (MEF) [4]. With regards to dynamic scenes, many MEF methods have been proposed, which can be broadly classified into conventional methods [5,6,7,8,9] and deep-learning-based (DL-based) methods [2]. In conventional methods, Sen et al. [5] and Hu et al. [10] use patch-based motion estimation to align the input images, and their performance is heavily affected by the accuracy of the motion estimation. Lee et al. [11] and Oh et al. [12] present iterative optimization frameworks based on low-rank minimization for obtaining fused images, which suffers from a high computational requirement. Methods such as [13,14] attempt to detect moving objects by analyzing the consistency of pixel intensities between the input images. However, the introduced intensity thresholds usually results in inaccurate motion estimation. Recent work has shown that deep-learning-based methods [15,16,17] often achieve state-of-the-art performance on various benchmark datasets.

Many DL-based methods often pre-align LDR images before fusing them with neural networks to deal with motion problems. Hence, the fusion results are strongly influenced by the alignment algorithms [18,19,20]. Due to inaccuracy in motion estimation, the aligned images will be distorted, resulting in artifacts in the HDR image fused by the neural networks. To reduce the influence of misalignment of the LDR images, other DL-based methods [15,21,22], by contrast, use direct feature concatenation to fuse the LDR images. Some of them introduce image correlation to guide the feature alignment [16,23], where the attention mechanism [24] is applied to exclude misaligned features. However, correlation-guided feature alignment is sensitive to over-saturated regions, often losing textual details. In addition, correlation attention cannot distinguish the different areas caused by over-/under-exposure and object motion, resulting in ghosting effects on the motion area.

Ideally, we expect a network to detect regions of differences between LDR images and determine whether these differences are the results of motion or saturation. The network should be able to fuse regions where saturation occurs and ignore texture differences caused by movement. Moreover, we need to avoid image distortion caused by motion estimation errors. To achieve that, we propose a motion-attention deep fusion network for HDR deghosting (DDFNet), which uses correlation and motion information to guide the merging of the LDR images and fuses HDR images without ghosting. Our main contributions can be summarized as follows:

- We demonstrate that motion information of the LDR images can distinguish the saturation area from the motion area of the LDR images. Hence, we propose to use motion information (e.g., optical flow) to guide the fusion of details in the foreground and background and to prevent ghosting of the HDR image.
- We propose an end-to-end attention-based fusion framework that uses a motion estimation module and a correlation estimation module to obtain the optical flow and image correlation clues, respectively. Then, the estimated optical flow, as well as the correlation clue, guide the network to pay more attention to the features from the saturation and motion areas by an attention-based fusion module and direct the network to reconstruct credible HDR details in the presence of saturation, occlusion, and underexposure.
- On both datasets with and without ground truth, we report stat-of-the-art fusion results.

## 2. Related Work

Deep-learning-based multi-exposure fusion (MEF) methods restore scene irradiance using feature learning from LDR images. Given a set of multi-exposure LDR images $X=\{x_{1},x_{2},\cdots ,x_{n}\}$, the deep-learning-based HDR imaging method aims to learn a mapping function *M* with parameters θ that maps the LDR counterparts to an HDR image *Y*:

(1) Y=M(X;θ)

The fused HDR image should have high bit depth, high contrast ratio, and preserved details [2]. In dynamic scenes, the HDR image also needs to be ghost-free.

Previous works adopt image alignment before merging them into the HDR image. For example, Kalantari et al. utilize an optical flow algorithm [19] to align low- and high-exposure images to the medium-exposure image defined as the reference image, then feed the aligned images to the deep neural networks (DNN) to generate the HDR image. A similar strategy is adopted by Yan et al. [25], where they extract features by multi-scale and compute multi-scale loss, which obtains enriched information of the HDR images. However, the classical optical flow methods tend to misestimate under conditions of large-scale motion and significant exposure differences. Therefore, Peng et al. replace the optical estimator with an optical flow network such as FlowNet [26], which still suffers distortion of aligned LDR images.

Instead of pre-alignment, many DL-based methods directly concatenate the encoded LDR image features and reconstruct the HDR image supervised by the ground-truth HDR image. One of the representative methods is DeepHDR [15], which constructs an encoder–decoder framework. DeepHDR abstracts features of the LDR images by their encoders and concatenates the encoded features, which are then sent to the decoder to reconstruct the HDR image. Although simple, this network can obtain better quality HDR images after training with ground-truth images. This methodology has been adopted by [21,22], whereas the network structures have been improved to extract more valuable features from LDR images. These end-to-end concatenation-based methods, however, generate less realistic details in highly saturated areas.

To retain more details of the saturated regions, Yan et al. [16] adopt an attention mechanism [24] in their fusion framework. They add attention modules in the encoder to compute the correlation between the under-/over-exposure image and the reference image (medium-exposure image), which are then fed to refine the extracted feature maps of the LDR images. This spatial correlation attention is also applied by [23], where they use inter-attention of the self-similarity of pixels in LDR images for feature alignment. These spatial correlation-guided methods can help exclude misaligned details of non-reference LDR images but also exclude the details in the saturation regions. We find accurate motion attention can help reduce the exclusion of saturated regions of non-reference LDR images by distinguishing the motion regions from the saturated region. Therefore, we propose a fusion network based on correlation- and motion-attention to fuse HDR images with enriching details and less ghosting effect.

## 3. Proposed Method

### 3.1. Pipeline

In this section, we describe the proposed DDFNet in detail. We aim to fuse a sequence of non-strictly aligned LDR images with large objects’ motion into a ghost-free HDR image containing rich details. Given a set of LDR images of a dynamic scene $S={\left\{I_{i}\right\}}_{i=1}^{N}$ with an arbitrary number of frames *N*, one of these images is selected as the reference image, denoted as $I_{r}$. The desired HDR image *H* is supposed to be aligned with the reference image $I_{r}$ and should contain the saturation texture from every LDR image. We also apply the gamma correction used in [16] that maps the original LDR image $I_{i}$ to the domain closer to what we perceive with our eyes. The corresponding corrected image $P_{i}$ is obtained by

(2) $$P_{i}=\frac{I_{i}^{\gamma }}{t_{i}}\;,\gamma >1\;,\forall i=1,2,\ldots ,N\;,$$

where $t_{i}$ denotes the exposure time of the LDR images in *S*, and γ equals to 2.2, as suggested in previous work [5], which is also the value adopted in most video standards. These corrected images unify the LDR images to the same range that helps estimate the motion between the reference $I_{r}$ and other LDR images. We concatenate images $I_{i}$ and $P_{i}$ along the channel dimension to obtain a 6-channel stack $X_{i}=\left[I_{i},P_{i}\right],i=1,\ldots ,N$ and use it as the input of the network to obtain the HDR image $\tilde{H}$ by the proposed DDFNet:

(3) $$\tilde{H}=F\left(X;\theta \right)\;,$$

where F(·) represents the proposed DDFNet and θ denotes the network parameters. In particular, we apply the image tone-mapping after the HDR image has been fused for an easier visual comparison:

(4) $$H=T(\tilde{H})\;$$

where T can be defined according to visual preference.

### 3.2. Network Structure

**Figure 1:**
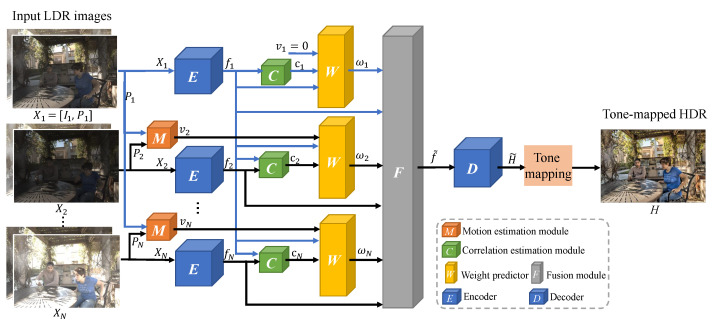
The framework of proposed DDFNet.

In our DDFNet, we use structure correlation between $I_{r}$ and other LDR images and the motion information to guide the merging of all LDR images. In contrast to traditional optical flow-based methods, where the HDR image is fused with motion-compensated LDR images, our method does not require motion compensation. Rather than pre-aligning images, our approach uses the optical flow as guidance to direct the network to focus on textured regions of different images and directly merge the raw LDR images to avoid ghosting caused by motion compensation errors. Figure 1 gives an overview of DDFNet’s architecture.

The network takes the concatenated LDR image and their gamma-corrected images as inputs ${\left\{X_{i}\right\}}_{i=1}^{N}$. The images are first encoded independently to obtain the corresponding deep feature representations ${\left\{f_{i}\right\}}_{i=1}^{N}$. Specifically, we define $I_{1}$ as the reference image and refer to the feature map $f_{1}$ encoded from the reference image pair $X_{1}=\left[I_{1},P_{1}\right]$ as the reference map, where $P_{1}$ is the gamma correction of image $I_{1}$ as introduced in Formula (2). For each image, the encoded feature map ${\left\{f_{i}\right\}}_{i=2}^{N}$ is then concatenated with the common feature map $f_{1}$ and sent to a correlation estimation module so that the structure correlation between the reference and other images can be estimated. Meanwhile, we concatenate the non-reference gamma-corrected images ${\left\{P_{i}\right\}}_{i=2}^{N}$ with the reference gamma-corrected image $P_{1}$, then feed them into a motion estimation module to obtain the optical flow $v_{i}$ of $P_{i}$ with respect to $P_{1}$. Next, a weight predictor computes element-wise fusion weights ${\left\{\omega_{i}\right\}}_{i=1}^{N}$ based on the encoded reference feature map $f_{1}$, the optical flow clue ${\left\{v_{i}\right\}}_{i=1}^{N}$, and the correlation information ${\left\{c_{i}\right\}}_{i=1}^{N}$. We propose an attention-based fusion approach, which combines the input with the predicted weights ${\left\{\omega_{i}\right\}}_{i=1}^{N}$ and creates the fused feature map $\hat{f}$. In this way, the network can adaptively select the most useful information from each LDR image. The merged $\tilde{f}$ is fed to the decoder module to produce the HDR image $\tilde{H}$. Our final output is tone-mapped to the HDR image *H* for better visualization.

#### 3.2.1. Encoder

We concatenate the RGB LDR image $L_{i}$ and corresponding gamma-corrected image $P_{i}$ along the channel dimension as the input $X_{i}\in R^{W\times H\times 6}$. The encoder *E* consists of two convolutional layers, each followed by a Rectified Linear Unit (ReLU). The convolutional kernels have the size of 3×3 and the stride of 1, and there are 64 kernels in each layer. The encoder independently maps each input $X_{i}$ to a deep feature representation $E\left(X_{i}\right)=f_{i}\in R^{W\times H\times 64}$, which is a rich embedding of the input images. The encoding for both LDR and gamma-corrected images helps identify the saturated regions. As our experiments demonstrate (Section 4.2), this gives much better results than using only the LDR image.

#### 3.2.2. Motion Estimation Module

One of the important challenges of dynamic HDR is the movement between frames, especially when the motion of objects is fast. It is difficult to determine if the different regions in LDR images result from the movement or saturation of different exposures. Using a motion estimation module, we propose utilizing motion attention to guide the network to obtain beneficial features from movement regions. Original LDR images have significantly different pixel intensities due to different exposure times, making it difficult to estimate the motion of two images. Hence, we use the gamma-corrected *P* as input, which has a uniform light intensity distribution and significantly improves the calculation of the optimal flow. When we sort the images by exposure time from short to long, we usually find the final HDR image is closer to the image in the middle. We take ⌊(N+1)/2⌋ as the image with median exposure time, where *N* is the total number of the LDR images. This median exposure image is the base frame or reference image, denoted as $I_{1}$. Then, every frame is sent along with the base frame to the motion estimation module to obtain a pixel-wise optical flow map. We use a well-established optical network PWC-Net [27] for flow estimation because of its high accuracy and speed. The estimated motion map ${\tilde{v}}_{i}\in R^{W\times H\times 2}$ is encoded by three convolutional layers, each followed by a ReLU, to obtain the motion feature maps $v_{i}\in R^{W\times H\times 64}$. The convolutional kernels have the size of 3×3 and the stride of 1. Each layer has 64 kernels. Then, we use the motion feature maps as the attention to reweigh the fusion weight maps. In this way, the network can retain the motion of an object as seen in the reference frame and gain information about the background from the other LDR images, thereby avoiding ghosts caused by inaccurate motion compensation.

#### 3.2.3. Correlation Estimation Module

We propose a correlation estimation module to introduce spatial attention, except for motion attention. The correlation estimation module *C* (CEM) comprises a convolutional layer and one ReLU layer, where the convolutional kernel has the size of 3×3 and the stride of 1, and there are 64 kernels. The encoded feature maps from one base image and another LDR image are concatenated and then fed into the CEM to yield correlation feature maps $c_{i}\in R^{W\times H\times 64}$. Note that the input of the top CEM in Figure 1 is the concatenation of two identical base images. These feature maps encode the structure correlation and exposure difference between two images, which helps the network to pay attention to the saturation area of different images and retain useful texture details from saturated regions.

#### 3.2.4. Fusion Module

The fusion module adaptively combines information across the individual LDR images to generate a merged feature map $\tilde{f}$. In principle, pooling operations such as max pooling or average pooling across different LDR images could be used to merge features. However, we found that pooling cannot provide satisfactory fusion results. Inspired by [28], we adopt an attention-based fusion approach that uses the motion features $v_{i}$ and correlation features $c_{i}$ as attention to predict the fusion weight and fuse the feature maps by a weighted sum. Specifically, we independently feed the reference feature maps $f_{1}$, the motion feature maps $v_{i}$, and the correlation feature maps $c_{i}$ to the weights predictor *w* composed of two ResNets [29] to obtain the attention weights $\tilde{\omega }$ by

(5) $$\tilde{w_{i}}=W\left(f_{1},v_{i},c_{i}\right)\;.$$

Note that the motion feature maps $v_{1}$ for the reference image are all equal to zero. The ResNet has 64 kernels with the size of 3×3 in each convolutional layer. Then, the output attention weight map $\tilde{w_{i}}\in R^{W\times H\times 64}$ has the same size as the encoded feature maps $f_{i}$. Then, the normalized attention weight of each input LDR is calculated by

(6) $$w_{i}\left(x,y\right)=\frac{\tilde{w_{i}}\left(x,y\right)}{\sum_{j=1}^{N}\tilde{w_{j}}\left(x,y\right)}\;,$$

where (x,y) is the coordinate index of pixels in the feature map. We can obtain the merged feature by

(7) $$\tilde{f}=\sum_{i=1}^{N}w_{i}\cdot f_{i}\;,$$

where · represents element-wise multiplication. The merged feature is then passed to the decoder to produce the fused HDR image.

#### 3.2.5. Decoder

The decoder module *D* generates the fused HDR from the fused feature $\tilde{f}$. We first project the input feature maps $\tilde{f}$ to a higher dimension space (e.g., 128 channels) by a convolutional layer to fully extract their features, then we pass the fused feature maps through three residual dense networks [30] with 2-dilated convolutions [31] to reconstruct the HDR image similar to [16]. Here, the channel growth rate of the dilated residual dense block is 32, and each block contains 5 convolutional layers. The parameters of the architecture in the decoder are listed in Table 1. Compared with ordinary residual networks, dense residual networks with dilated convolutions can retain significant features after deep layers and have a broader view that aggregates information locally and globally, obtaining HDR images with a consistent intensity distribution and precise details.

### 3.3. Training Details

Since the HDR images are usually displayed after tone-mapping, we use a μ-law to compress the HDR image to a tone-mapped image:

(8) $$T\left(H\left(x,y\right)\right)=\frac{\log\left(1+\mu \tilde{H}\left(x,y\right)\right)}{\log(1+\mu )}\;,$$

where $\tilde{H}$ is the network’s output. *H* denotes the tone-mapped HDR image, which is also used for comparison with the ground-truth tone-mapped HDR image. We keep *H* in range [0,1] and set μ=5000. The $\ell_{1}$-norm is used as a loss function to minimize the difference between the tone-map-generated and ground-truth HDR images, which is defined as:

(9) $$L={∥T\left(H\right)-T\left(\hat{H}\right)∥}_{1}\;,$$

where $\hat{H}$ is the ground-truth HDR image. We train the network on the HDR dataset [18] that contains 74 training samples with LDR images and the corresponding ground-truth HDR image. The training images have a resolution of 1500×1000 and are augmented with cropping, rotating, and folding. A total number of $1.11\times 10^{6}$ cropped image patches with the size of 128×128 are used during training, enabling the network to learn about various image textures.

Our DDFNet is implemented by Pytorch on a CPU of Inter Xeon 39 GB RAM and a GPU of NVIDIA Tesla V100. The model is trained with an Adam optimizer. We apply a batch size of 2 and total epochs of 5000. The multi-step learning rate (LR) decay is adopted, where the LR starts at $1\times 10^{-5}$ and is reduced by half when the number of epochs reaches 4000 and 4500. The filter size is set to 3×3 for all convolutional layers, and the stride size is 1×1. Note that we use a trained PWC-net as the optical flow estimation module, which is frozen at the beginning and trained together with the whole network after 4000 epochs. In this way, the motion estimation module can take advantage of the original trained PWC-net to estimate optical flow and adapt to the HDR fusion task after the fine-tune.

## 4. Experimental Results

### 4.1. Comparison with the State of the Art

In this section, we take visual and objective comparisons of the proposed method and other state-of-the-art methods.

#### 4.1.1. Comparison on Dataset with Ground-Truth

First, we conduct experiments on the dataset in [18], which contains 15 test samples. Every test sample has three LDR images and one ground-truth HDR image with a size of 1500×1000. To evaluate the objective quality of the fused HDR image, we compute PSNR and SSIM for images after tone-mapping using a μ-law (PSNR-μ/SSIM-μ) and linear domains (PSNR-*L*/SSIM-*L*). Furthermore, we use HDR-vdp2 [32] to evaluate the HDR images. The HDR-vdp2 is based on a new visual model for all luminance conditions which has been derived from new contrast sensitivity measurements. A higher HDR-vdp2 value indicates a better HDR fusion result.

Table 2 illustrates the quantitative comparison of our method against the state of the art, including the patch-based method [5], three DL-based methods with alignment [15,17,18], and a DL-based method without pre-alignment [16]. All results are reproduced on the corresponding released code ([5] https://web.ece.ucsb.edu/psen/hdrvideo,^15^] https://elliottwu.com/projects/18_hdr/,^16^] https://donggong1.github.io/ahdr.html,^18^] https://cseweb.ucsd.edu//viscomp/projects/SIG17HDR/ (accessed on 22 September 2022)). Note that we adopt the results (PSNR-μ and PSNR-*L*) from Prabhakar’s paper [17] since the code is unavailable. As we can see, Kalantari [18], DeepHDR [15], and Prabhaker [17] use pre-alignment based on optical flow or homographic transformation. Additionally, boundary cropping is used after the alignment based on the optical flow in [17,18].

**Figure 2:**
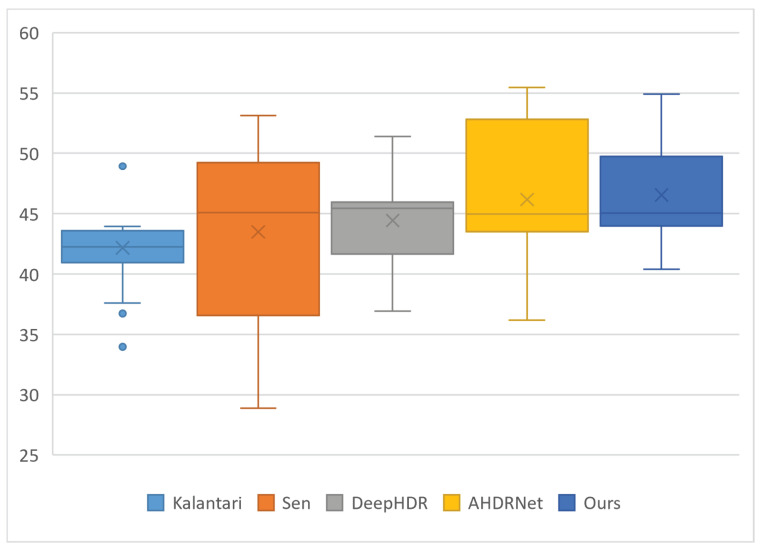
Boxplot of PSNR-μ of the fused images generated by Kalantari [18], Sen [5], DeepHDR [15], AHDRNet [16], and our method, respectively.

Without any pre-alignment, our method achieves the best results in PSNR-μ, SSIM-*L*, and HDR-vdp2, while achieving the second best results in SSIM-μ and PSNR-*L*. The analysis based on PSNR-μ (Figure 2) shows that our method does not only obtain the highest average PSNR-μ value but also has a lower degree of dispersion on PSNR-μ values, which means our method is robust to LDR images from different sources.

**Figure 3:**
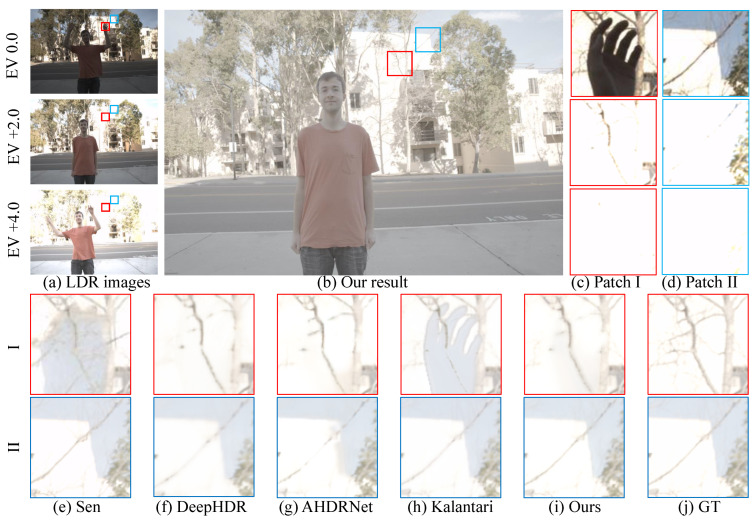
Comparison against the state-of-the-art methods on Kalantari’s dataset [18]. In the upper half of the figure, the left column (**a**) shows the LDR images with three exposure levels, (**b**) is our tone-mapped HDR result, and the last two columns (**c**,**d**) show two zoomed-in LDR regions (I and II) marked in the HDR image. In the lower half, we compare the zoomed-in HDR regions (I and II) of our results (**i**) with other results [5,15,16,18] (**e**–**h**), and GT represents the zoomed-in ground truth (**j**).

Figure 3 shows a sample of Kalantari’s dataset [18]. This dataset includes three LDR images with low-, middle-, and over-exposure levels. We compare the zoomed-in patch of the fused images tone-mapped by μ-low. Figure 3 shows that Sen [5,18] suffers from a ghosting effect on the zoomed-in region I due to the moving hand, whereas our method does not. While DeepHDR [15] and AHDRNet [16] avoid the ghosting problem, they also exclude texture details of tree branches in region II. On the other hand, our method successfully fuses the details from three LDR images and reconstructs the HDR image with a distinct structure, such as the edge of the wall.

#### 4.1.2. Comparison on Dataset without Ground-Truth

**Figure 4:**
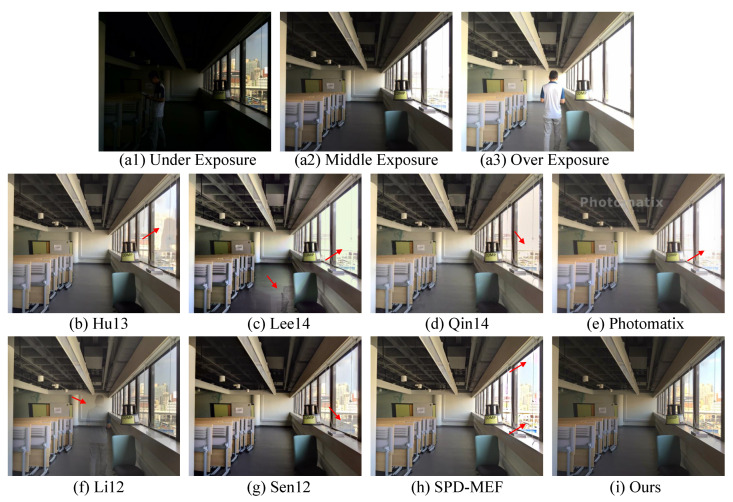
An example of a multi-exposure source image sequence (**a1**–**a3**) and fused images (**b**–**i**) generated by [5,10,11,14,33,34,35] and our method, respectively.

**Figure 5:**
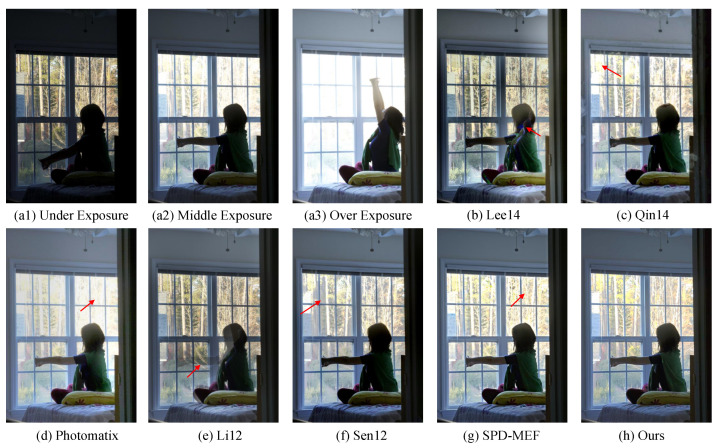
An example of a multi-exposure source image sequence (**a1**–**a3**) and fused images (**b**–**h**) generated by [5,11,14,33,34,35] and our method, respectively.

We also compare our method with other MEF fusion methods [5,10,11,14,33,34,35] for dynamic scenes on the dataset in [4], which includes 20 samples with at least three LDR images but without the ground-truth HDR image. The subjective comparisons are shown in Figure 4. We can see that the under-exposure LDR image loses image detail inside the room, while the middle- and over-exposure images lack texture outside the window. Methods, such as those in [11,33,34], miss the most information from the under-exposure on the window region. Although Hu13 [10], Sen [5], and SPD-MEF [14] retain part of the information of the outside, they suffer from unpleased artifacts. Additionally, ghosting effects occur in Lee14 [11] and Li12 [35]. In contrast, our method can not only retain information from all LDR images but also avoid ghosting caused by moving objects. Similarly, Figure 5 shows that our method preserves more details of the background.

### 4.2. Further Analysis

Here, we investigate the impact of different components in the proposed DDFNet architecture. All analyses are conducted on Kalantari’s dataset [18] because it includes the ground-truth HDR images for objective comparison.

#### 4.2.1. Analysis of Network Structure

Here, we analyze the impact of different modules in our framework. (a) **No Attention Module**: This framework is a full encoder–decoder network without any attention module, where the corresponding decoder encodes input LDR images. Then, the encoded feature maps are concatenated along the channel dimension and fed into the decoder to reconstruct the HDR image. (b) **Only Correlation Attention**: This framework contains the correlation estimation modules that generate the correlation feature maps for the guidance of fusion weight computing. (c) **Only Motion Attention**: This structure only takes the motion information for attention to guide the fusion weight computing. (d) **Correlation + Motion + Warp**: This framework contains the correlation estimation and motion estimation modules at the same time. However, the output optical flow of the motion estimation module is used to warp the non-reference feature maps based on the reference feature. Then, the warped feature maps of the LDR images, the correlation feature maps generated by the correlation estimation module, and the reference feature maps are fed into the weights predictor to compute the fusion weights for each LDR image. (e) **Correlation + Motion + Attention (Ours)**: Rather than warp the feature maps of the LDR images, the output of the motion estimation module is directly used as the attention for the prediction of the fusion weights.

Apart from the differences mentioned in the network structure, all other aspects of the above five network structures remain the same, as does the training strategy. The results can be seen in Table 3.

**Figure 6:**
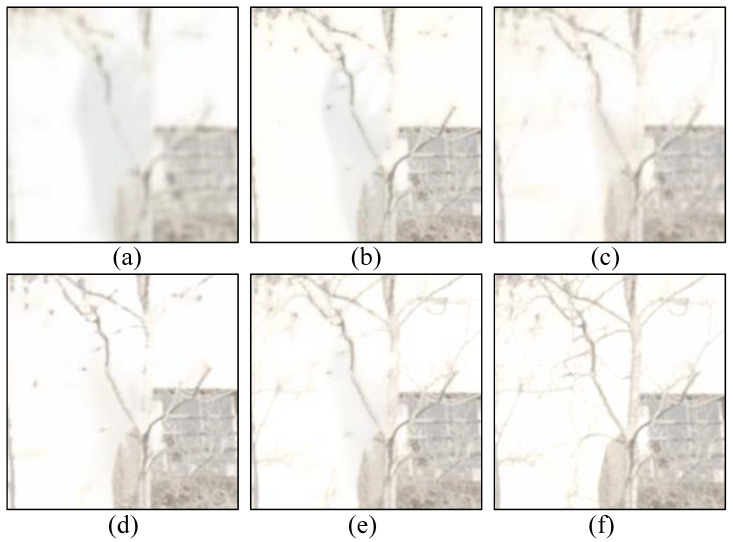
Comparison of the fused results from different network structures with (**a**) no attention module, (**b**) only correlation attention, (**c**) only motion attention, (**d**) correlation + motion + warp, and (**e**) correlation + motion + attention. The last image (**f**) represents the ground truth. The zoomed-in patches are shown to make a better comparison.

We find that the framework without any attention module (a) obtains poor results, indicating that a simple encoder–decoder architecture is insufficient to fuse useful information in dynamic scenes. In contrast to the structure without attention mechanisms, the correlation attention (b) as well as the attention based on motion information (c) can significantly improve the quality of the fused image, among which correlation attention (b) provides an even more substantial performance improvement than motion attention (c). While structure (e) contains the same motion estimation module as (d), it uses motion information to guide calculations of fusion weights, which helps improve HDR quality as compared with the structure (d), which warps the feature map based on estimated optical flow. Additionally, Figure 6 illustrates the efficiency of motion attention for ghosting removal (c–e) compared with using only correlation attention (b).

#### 4.2.2. Analysis of Encoder

We analyze the impact of the different encoders in our framework. (a) **LDR image**: This framework only uses the original LDR images $L_{i}$ for the encoder to generate the LDR feature maps, which are fed into the correlation estimation module and then are adaptively merged by the fusion module. (b) **LDR and gamma correction (GC) images**: This framework concatenates the original LDR images $L_{i}$ and the corresponding gamma correction images $P_{i}$ by Equation (2) along the channel dimension and encodes them as the LDR feature maps for image fusion. As shown in Table 4, the encoding for both LDR images and the corresponding gamma correction images can significantly improve the fusion results compared with encoding the LDR images. When gamma correction is applied, the pixel values of images with different exposures are corrected to the same range. Consequently, it is easier for the network to determine if two images are structurally different because of over-exposure or under-exposure, allowing the network to retain more details on the fusion images.

#### 4.2.3. Image Fusion for Denoising

In practical applications, the acquired LDR images are not as ideal as the LDR images in the database, which usually contain noise. The network often retains noise as useful information, resulting in poor quality of fused HDR images. To reduce the effect of noise on the fused HDR images, we fine-tune the trained model with a denoising task. Specifically, we adopt the same training dataset as the basic model. The difference is that we add an i.i.d. Gaussian noise in random proportions for each input LDR image. The network is fine-tuned for around 1000 epochs until it converges again.

**Figure 7:**
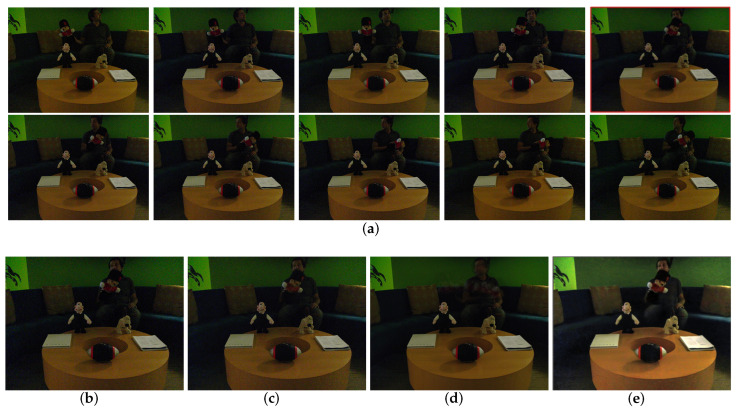
Image fusion for denoising. (**a**) Input images. (**b**) Reference input. (**c**) Sen [5]. (**d**) Li12 [35]. (**e**) Ours.

As shown in Figure 7a, the input is a sequence of images with noise and moving objects. We take the 5th noise image as reference and fuse all images by the methods of Sen [5] (Figure 7c), Li12 [35] (Figure 7d), and our fine-tuned networks (Figure 7e). As can be seen, the output of Sen [5] is ghosting-free. However, it is full of noise. Li12 [35] obtains a fused image with lower noise but suffers from severe motion ghosting in the result. In contrast, our method suppresses most of the noise and fuses the image to be ghosting-free.

#### 4.2.4. Image Fusion for Traffic Scenes

**Figure 8:**
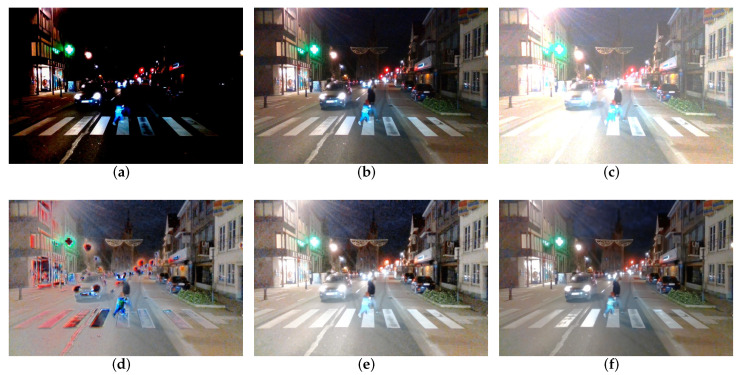
Image fusion for traffic scene. (**a**) Under-exposure image. (**b**) Middle-exposure image. (**c**) Over-exposure image. (**d**) Fusion by AHDRNet [16]. (**e**) Fusion by our basic network. (**f**) Fusion by fine-tuned network.

We apply our fine-tuned network in traffic scenes as shown in Figure 7. This image set was taken in the city center of Ghent, which contains three LDR images with different exposures from under to over (Figure 8a–c). With poor lighting conditions, the captured images contain a lot of noise. Additionally, the pedestrians and vehicles in the images have movement between two LDR images due to the time differences in camera shooting. We apply a state-of-the-art DL-based fusion method, AHDRNet [16], to fuse three images, which obtains HDR images with severe distortion (Figure 8d). This distortion is a specific problem in applying deep-learning-based methods: the trained network only works on data that are precisely identical to the training data and degrades seriously when used on the real scene. Although adopting the same training data as AHDRNet [16], our method can generalize to the actual scene and obtain a better fusion image as in Figure 8e. However, our basic network suffers from the noise problem while fusing the original images, which is solved by our fine-tuned network (Figure 8f). As can be seen, the fused image contains more details of the vehicle and the street scene. The reconstructed HDR image can help computer vision tasks such as pedestrian detection, traffic light detection, etc.

#### 4.2.5. Limitation of DDFNet

**Figure 9:**
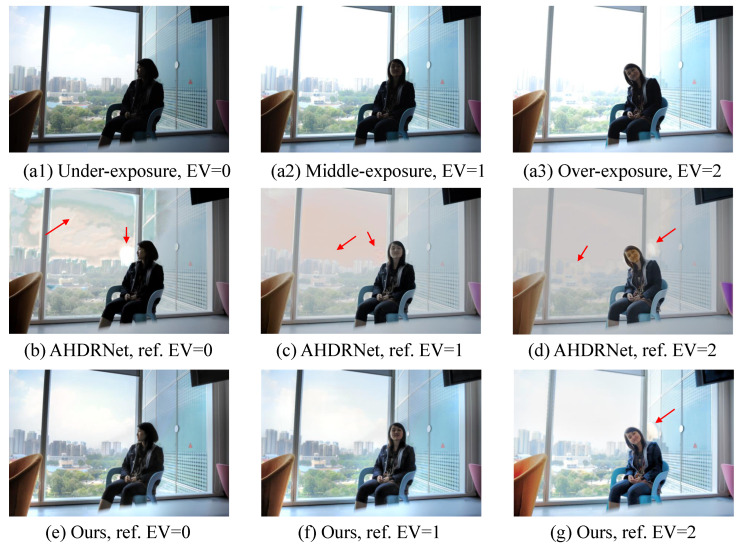
An example of a multi-exposure source image sequence (**a1**–**a3**) and fused images generated by the method in [21] (**b**–**d**) and our method (**e**–**g**) using different exposure images as references.

As described in Section 3.2.2, we choose the LDR image with median exposure time as the reference image. In certain extreme cases, our proposed DDFNet method produces halo artifacts, as shown in Figure 9g. This is because when using the over-exposure image as the reference, the region reconstructed into a halo is marked as a motion region, where no background information can be obtained on the other exposures. In contrast, the fused images with under-/middle-exposure images as reference do not have this problem (Figure 9e,f). Consequently, the choice of the reference image is important for DDFNet, just as for the other DL-based methods [21]. For this problem, we can refer to the [36], which uses block-level template matching to fill holes. In addition, image in-painting [37] can also be considered for DL-based HDR imaging.

## 5. Conclusions

In this paper, we propose an effective HDR deghosting fusion network (DDFNet) driven by motion attention and image correlation attention. Specifically, the DDFNet estimates the optical flow of the LDR images using a motion estimation module and encodes that optical flow as flow features. In addition, it extracts correlation features between the reference LDR and other LDR images. In an attention-based fusion module, the optical flow and correlation features are used to combine information from LDR inputs adaptably. Following the merging of features, a Dense Network decoding algorithm reconstructs the HDR image without ghosting. The experimental results show that the proposed DDFNet outperforms other state-of-the-art models on several datasets. We also analyzed the contribution of different components of the proposed framework and demonstrated the efficiency of our motion-attention algorithm. This field of research still faces many challenges. The development of robust neural networks, for instance, requires a large number of labeled datasets. For multi-exposure HDR imaging, cameras and equipment are relatively expensive. Consequently, data-efficient learning needs to be investigated in future research. Alternatively, semi-supervised learning [38] or knowledge transfer [39] might be promising directions for DL-based HDR imaging. In the future, we plan to explore our method’s efficient and practical applications on consumer electronic products such as mobile photography.

## Figures and Tables

**Table 1:** Architecture of the decoder, where Conv represents the convolutional layer. The last two columns represent the number of input and output channels, respectively.

Block	Layer	Filter Size	Dilation	Padding	Input	Output
Conv_layer	Conv + ReLU	3×3	1	1	64	128
Conv_layer	Conv + ReLU	3×3	1	1	128	64
DenseBlock 1	Conv	3×3	2	2	64	32
	Conv	3×3	2	2	96	32
	Conv	3×3	2	2	128	32
	Conv	3×3	2	2	160	32
	Conv	3×3	2	2	192	32
Conv_layer	Conv	3×3	1	1	224	64
DenseBlock 2	Conv	3×3	2	2	64	32
	Conv	3×3	2	2	96	32
	Conv	3×3	2	2	128	32
	Conv	3×3	2	2	160	32
	Conv	3×3	2	2	192	32
Conv_layer	Conv	3×3	1	1	224	64
DenseBlock 3	Conv	3×3	2	2	64	32
	Conv	3×3	2	2	96	32
	Conv	3×3	2	2	128	32
	Conv	3×3	2	2	160	32
	Conv	3×3	2	2	192	32
Conv_layer	Conv	3×3	1	1	224	64
Tail	Conv	3×3	1	1	192	64
	Conv	3×3	1	1	64	64
	Conv + ReLU	3×3	1	1	64	3

**Table 2:** Quantitative comparisons of our method with state-of-the-art methods on Kalantari dataset, with the best result in bold and the second-best result underlined. Here, O.F. and Homo. refer to the optical-flow-based alignment and the homographic transformation adopted by different methods, respectively.

Methods	Pre-Alignment		Boundary Cropping	PSNR-μ	SSIM-μ	PSNR-*L*	SSIM-*L*	HDR-vdp2
	O.F.	Homo.						
Sen [5]				43.49	0.9860	40.14	0.9764	65.58
Kalantari [18]	✓		✓	42.17	0.9828	42.26	0.9841	67.88
DeepHDR [15]		✓		44.44	0.9917	**44.01**	0.9902	62.82
Prabhakar [17]	✓	✓	✓	42.82	-	41.33	-	-
AHDRNet [16]				46.16	**0.9927**	43.24	0.9901	68.46
Ours				**46.53**	0.9924	43.38	**0.9916**	**69.17**

**Table 3:** Impacts of different modules for the fusion framework, where Corre. and Atten. are abbreviations of Correlation and Attention, respectively. The last structure (e) is adopted in our method.

Modules	PSNR-μ	SSIM-μ	PSNR-*L*	SSIM-*L*	HDR-vdp2
(a) No Attention	40.38	0.9886	39.75	0.9813	67.50
(b) Only Corre. Attention	46.38	0.9918	43.35	0.9915	68.45
(c) Only Motion Attention	45.50	0.9911	42.52	0.9892	68.09
(d) Corre. + Motion + Warp	46.39	0.9922	43.33	0.9915	68.48
(e) Corre. + Motion + Atten.	**46.53**	**0.9924**	**43.38**	**0.9916**	**69.17**

**Table 4:** Comparison between our approach for encoding both LDR images and their gamma correction (GC) images and a baseline approach that only encodes the LDR image.

Encoded	PSNR-μ	SSIM-μ	PSNR-*L*	SSIM-*L*	HDR-vdp2
(a) LDR	43.42	0.9890	40.32	0.9834	67.32
(b) LDR + GC	**46.53**	**0.9924**	**43.38**	**0.9916**	**69.17**

## Data Availability

No new data were created or analyzed in this study. Data sharing is not applicable to this article.
